# A Novel Brain Network Construction Method for Exploring Age-Related Functional Reorganization

**DOI:** 10.1155/2016/2429691

**Published:** 2016-02-29

**Authors:** Wei Li, Miao Wang, Yapeng Li, Yue Huang, Xi Chen

**Affiliations:** ^1^College of Automation, Huazhong University of Science and Technology, Wuhan 430074, China; ^2^Image Processing and Intelligent Control Key Laboratory of Education Ministry of China, Wuhan 430074, China; ^3^School of Electrical and Electronic Engineering, East China Jiaotong University, Nanchang 330013, China

## Abstract

The human brain undergoes complex reorganization and changes during aging. Using graph theory, scientists can find differences in topological properties of functional brain networks between young and elderly adults. However, these differences are sometimes significant and sometimes not. Several studies have even identified disparate differences in topological properties during normal aging or in age-related diseases. One possible reason for this issue is that existing brain network construction methods cannot fully extract the “intrinsic edges” to prevent useful signals from being buried into noises. This paper proposes a new subnetwork voting (SNV) method with sliding window to construct functional brain networks for young and elderly adults. Differences in the topological properties of brain networks constructed from the classic and SNV methods were consistent. Statistical analysis showed that the SNV method can identify much more statistically significant differences between groups than the classic method. Moreover, support vector machine was utilized to classify young and elderly adults; its accuracy, based on the SNV method, reached 89.3%, significantly higher than that with classic method. Therefore, the SNV method can improve consistency within a group and highlight differences between groups, which can be valuable for the exploration and auxiliary diagnosis of aging and age-related diseases.

## 1. Introduction

Healthy aging, along with many age-related diseases, is generally accompanied by cognitive functional deficits, such as reduced performance in memory and motor execution [1, 2], resulting from abnormalities in brain's structural and functional systems [3, 4]. Previous studies have illustrated that, in terms of structural changes, such functional degradations are related to loss of gray matter and thinning of cerebral cortex [5, 6]. Researchers are also currently attempting to explore aging from the perspective of alterations in functional system [7–9]. Owing to its noninvasive and mature data acquisition and processing, resting-state functional magnetic resonance imaging (fMRI) technology has become an important means to study these functional changes in the brain. fMRI has been providing abundant lines of evidence demonstrating variations in brain function during aging [4].

Since Watts and Strogatz [10] proposed small-world network in 1998 and Barabasi and Albert [11] proposed scale-free network in 1999, complex network theory has become increasingly important in exploring the nature of complex systems. Studies on the complex network of the brain, which is one of the most complex systems, elucidate the brain connectivity on the level of network topological organization, providing new insights into brain complexity [12, 13]. Numerous meaningful results have been obtained from exploring the changes in structural [3, 14] and functional brain networks [2, 15–17] of aging, as well as of age-related diseases, such as Alzheimer's disease (AD), Parkinson's disease, and stroke [18, 19]. Functional connectivity focuses on the relationship among different brain regions [20] and is usually established by the correlation of blood oxygenation level-dependent time courses between nodes [21]. Pearson correlation analysis, being the most frequently used functional brain network construction method, has been widely applied to explore the brain mechanism of aging and age-related diseases [7, 8, 22–24].

Numerous studies have demonstrated that reorganization in brain networks and changes in brain connectivity occur during aging [4, 19]. Alterations in brain network properties, such as efficiency and clustering coefficient, are also observed in aging [15, 16, 25, 26]. However, the consistency of the graph properties of brain networks within a group is sometimes not very satisfactory [27]. Some scientists cannot obtain statistically significant differences in some graph properties between different groups. Some studies even got disparate or contradicting changes in normal aging or age-related diseases [19, 28]. For example, Achard and Bullmore [25] found a higher global efficiency but no significantly different local efficiency was observed in the young group; this result is inconsistent with those obtained by Wu et al., indicating that the young group showed lower global efficiency but higher local efficiency than the elderly group [26]. Moreover, in terms of age-related diseases, such as AD, Stam et al. [29] showed that, compared with the healthy control group, the average path length of AD patients is longer, and the clustering coefficient does not change. However, Supekar et al. [30] showed that, compared with the healthy control group, AD patients demonstrate lower clustering coefficient, whereas the average shortest path does not significantly change. In addition, Zhao et al. [24] found a significant difference in clustering coefficient between AD patients and healthy controls, whereas Sanz-Arigita et al. [31] could not obtain significant changes. Given that the reproducibility of fMRI datasets cannot be guaranteed [32], the study results bear much uncertainty. Some studies have shown that certain topological properties of functional brain network of the same group at two different time periods collected in the same test set also exhibit differences [33, 34].

The reasons for the abovementioned problems are possibly the individual differences not related to research factors we investigated within the group, as well as the differences in data preprocessing (such as band filtering and noise removal methods) [33, 34]. Moreover, the existing network modeling methods cannot fully extract significant differences between groups. Therefore, finding methods to improve SNR of data collection, to extract meaningful features from the collected datasets, and to explore new brain network construction methods will be of great significance for brain network analysis to strengthen consistency within group and highlight the differences between groups. Scientists have started working on this endeavor. For example, Liang et al. [34] reported that different filter bands affect the consistency of brain network analysis; meanwhile, Braun et al. [33] illustrated that the processing methods of MRI data possibly affect the reliability and robustness of brain network.

This paper proposes a novel brain network construction method, namely, subnetwork voting (SNV) method with sliding window, to explore age-related functional reorganization. This novel brain network modeling approach aims to reduce the impact caused by individual differences within group in brain network analysis and consequently weaken the differences within group and highlight the differences between groups. We hope that this method can help improve the reliability of brain network analysis in exploring aging and related diseases.

## 2. Materials and Methods

### 2.1. Subjects

A dataset of 28 right-handed healthy adults, including 14 young adults (6 males, 8 females; mean age: 23.71 years old; range: 19–30 years old) and 14 elderly adults (7 males, 7 females; mean age: 67.57 years old; range: 60–79 years old), was collected by the International Consortium for Brain Mapping (ICBM), which is a subdataset in the 1000 Functional Connectomes Project (http://fcon_1000.projects.nitrc.org/).

### 2.2. Data Preprocessing

The first five images in raw data of each subject were discarded by ICBM to ensure magnetization equilibrium. Data preprocessing was performed using SPM8 (http://www.fil.ion.ucl.ac.uk/spm/). We first conducted slice timing; that is, all the datasets were corrected in time domain. Realignment was subsequently employed to remove movement artifact in the fMRI time series. We then normalized the datasets by using a standard template. The processed images were smoothed by using a standard 4*∗*4*∗*4 FWHM kernel, drifted, and filtered to the frequency range of 0.06–0.11 Hz [12, 17]. The dataset for each subject was then segmented into 90 brain regions defined by AAL atlas [35].

### 2.3. Classic Construction Method for Functional Brain Network

For the time courses of 90 regions extracted from each subject, Pearson correlation was used to calculate the relationship between every two time courses of brain regions; thus, the correlation matrix of 90*∗*90 of each subject can be obtained. Previous studies have indicated that the properties of functional brain network are consistent with the actual brain model when the functional brain network density is 8–16% [7, 8, 17, 36]. Therefore, the functional brain networks of the young and the elderly individuals were established at a network density range of 8–16% to compare the topological properties (global efficiency, mean clustering coefficient, transfer coefficient, small-world value, and number of long edges) of the functional brain networks of young and old individuals in a large range of network densities.

### 2.4. SNV Method to Construct Functional Brain Network

In this study, we proposed the use of SNV method to establish functional brain network. The specific steps are as follows.

We set the density of functional brain networks to ND%; the length of time courses obtained by data preprocessing was *L* and the width of the sliding window was *W* (*W* < *L*).

**Figure 1:**
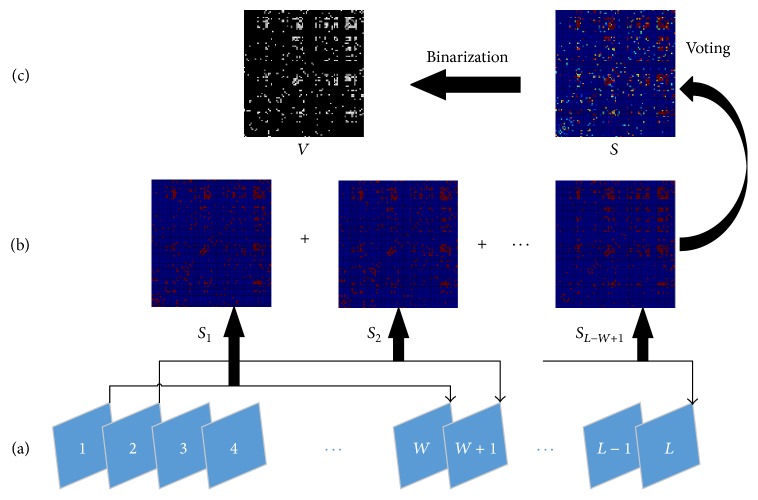
Process in the SNV method. (a) *L* − *W* + 1 subseries with length *W* were extracted through window sliding; (b) binary subnetwork matrix *S*_*i*_ (*i* = 1,2,…, *L* − *W* + 1) with density ND% was obtained by subseries; (c) the subseries were then summed up to obtain the voting matrix *S*, which was fixed to the binary voting network matrix *V* with density of ND%.

For the time courses with length *L* of 90 brain regions extracted from the preprocessing datasets of each subject, we first extracted *L* − *W* + 1 subseries with length *W* through window sliding (Figure 1(a)).

For all the *i*th (*i* = 1,2,…, *L* − *W* + 1) subseries of the 90 brain regions of each subject (these subseries were extracted from the original time courses with the same starting and ending time points), Pearson correlation method was used to calculate 90*∗*90 correlation coefficient matrix *C*_*i*_ (*i* = 1,2,…, *L* − *W* + 1), resulting in the construction of a brain subnetwork. According to the descending absolute value, the top ND percent elements of matrix *C*_*i*_ were set to 1, and the remaining elements were set to 0. As a result, an entitled binary subnetwork matrix *S*_*i*_ (*i* = 1,2,…, *L* − *W* + 1) with density ND% was obtained (Figures 1(a) and 1(b)).

We summed up all binary matrices *S*_1_, *S*_2_, *S*_3_,…, *S*_*L*−*W*+1_ and obtained a voting matrix *S* = *S*_1_ + *S*_2_ + *S*_3_ + ⋯+*S*_*L*−*W*+1_ (Figure 1(b)). Given that the functional brain network was established with density ND%, the top ND percent elements of matrix *S* (whose value is between 0 and *L* − *W* + 1) were set to 1, and the remaining elements were set to 0. We obtained the final desired binary voting network matrix *V* (Figure 1(c)), which describes the brain connectivity.

When more than one element is equal to the last one of the top ND percent element in *S* (whose value was *T*), then determining which elements should be set to 1 only depending on the voting matrix is difficult. We then used weight information of subnetwork sequence to make a decision. We calculated the absolute value of all elements in *C*_*i*_ and summed up these values to obtain *C* = |*C*_1_| + |*C*_2_| + ⋯+|*C*_*L*−*W*+1_|. Among the elements in *S* whose values are equal to *T*, we chose the ones demonstrating the largest absolute value with the same position in *C*; these values represent the largest weight of connectivity and thus were set to 1.

The length of a time course in our study is 128. To obtain the best values of window width in our modeling approach, we constructed the brain networks by using the SNV method with a window width of 10–120 and found that the rational window width is 90–100. Therefore, the experiments in our study were performed based on *W* = 90,95, and 100. We will give reason for the selection of window width and discuss this issue in Section 4.

### 2.5. Support Vector Machine (SVM) for Classification

**Figure 2:**
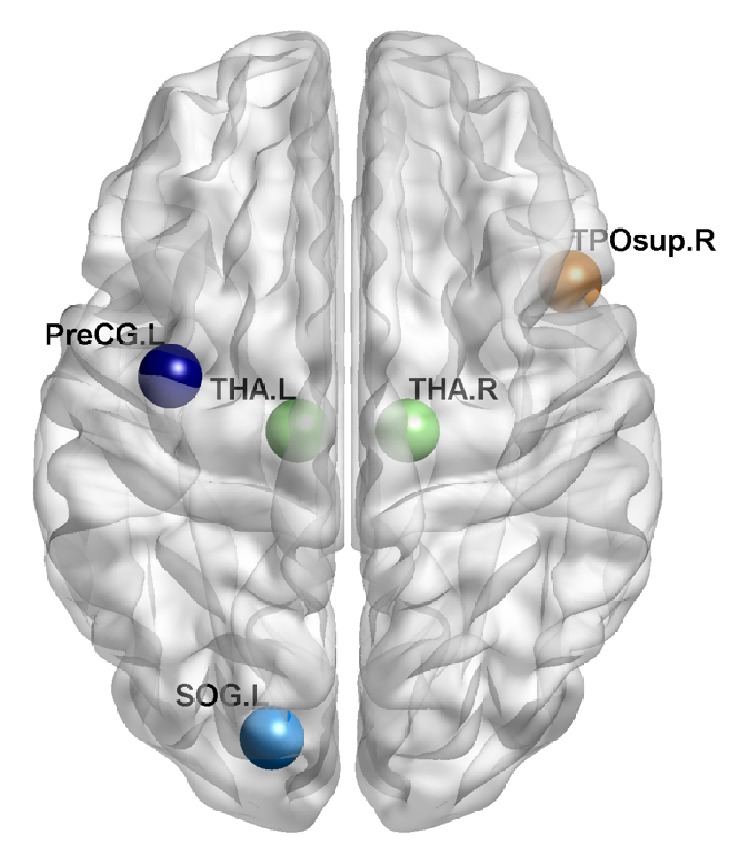
Regions showing significant difference in node degree. Different colors represent different clusters to which the nodes belong. Dark blue: parietal-(pre)motor; light blue: occipital; green: subcortical; brown: medial temporal.

This study used the SVM approach to classify the young and the elderly subjects to evaluate the SNV method in functional brain network construction. *K*-fold cross-validation was applied herein. Specifically, we randomly split the entire population into 14 folds, each consisting of one young and one elderly individual. We used 13 folds as training sample set, leaving one fold as testing sample set. The training and testing processes looped 14 times with a distinct testing fold in each time. The accuracy, sensitivity, and specificity were averaged across the 14 classifications. A linear kernel was applied in SVM, and two sets of classification features were considered in our experiments. The first set consisted of all the five network properties mentioned above. The other set, based on the first set, was optimized with nodal information and removed network properties showing no significant difference between groups (only the small-world value was removed according to this criterion). A two-sample *t*-test was performed to compare the node degrees of each node between the young and elderly groups in the training sample set, and those showing significant difference (FDR correction at *q* value of 0.05) were appended to the feature set. The nodes demonstrating significant difference based on SNV method were shown in Table 1 and Figure 2. The features were scaled from 0 to 1 before inputting them into SVM in both cases.

## 3. Results

### 3.1. Differences in the Topological Properties between the Young and Elderly Adults

**Figure 3:**
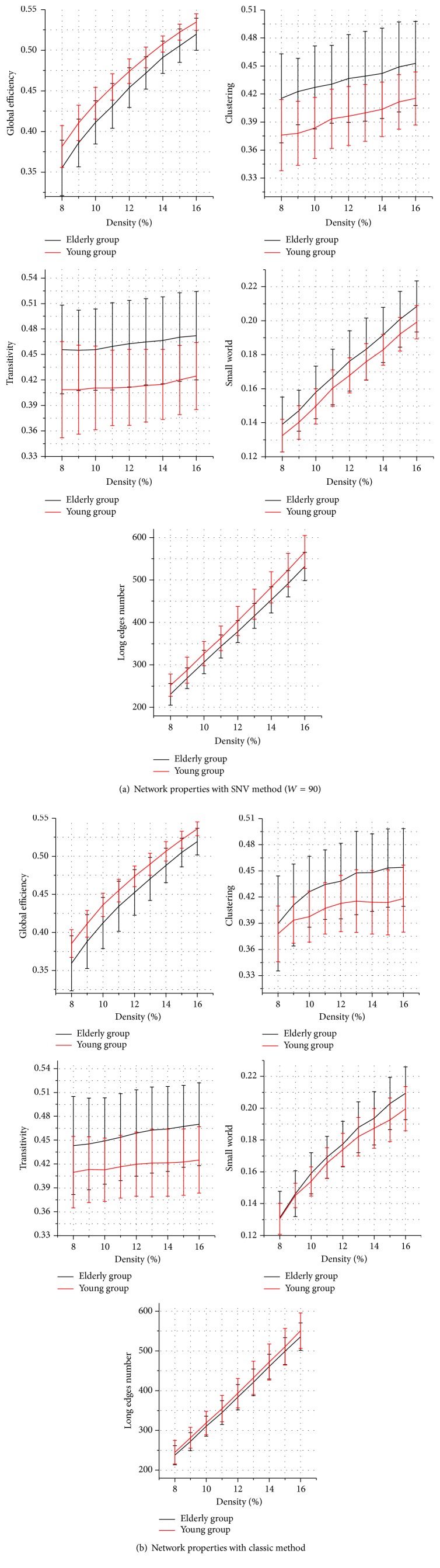
Topological properties of networks constructed by (a) SNV method (*W* = 90) and (b) classic method. The black and red lines represent the elderly and young groups, respectively. The error bars represent standard deviation. The difference between the networks, which were constructed by the SNV method, of the young and elderly groups remained consistent with the classic method.

With a network density of 8–16%, the topological properties of functional brain network in young and elderly groups were calculated based on classic method and SNV method (in this study, *W* = 90, 95, and 100), respectively, as shown in Figure 3 (take *W* = 90, e.g.). Compared with the topological properties of functional brain networks of young group, the global efficiency and the number of long edges of elderly group both declined to different extent. In addition, the mean clustering coefficient, transfer coefficient, and small-world property showed different degrees of increase. The difference between the brain functional networks, which are constructed by the SNV method, remained consistent with that by the classic method.

### 3.2. Statistical Analysis of Topological Properties of Brain Networks

**Figure 4:**
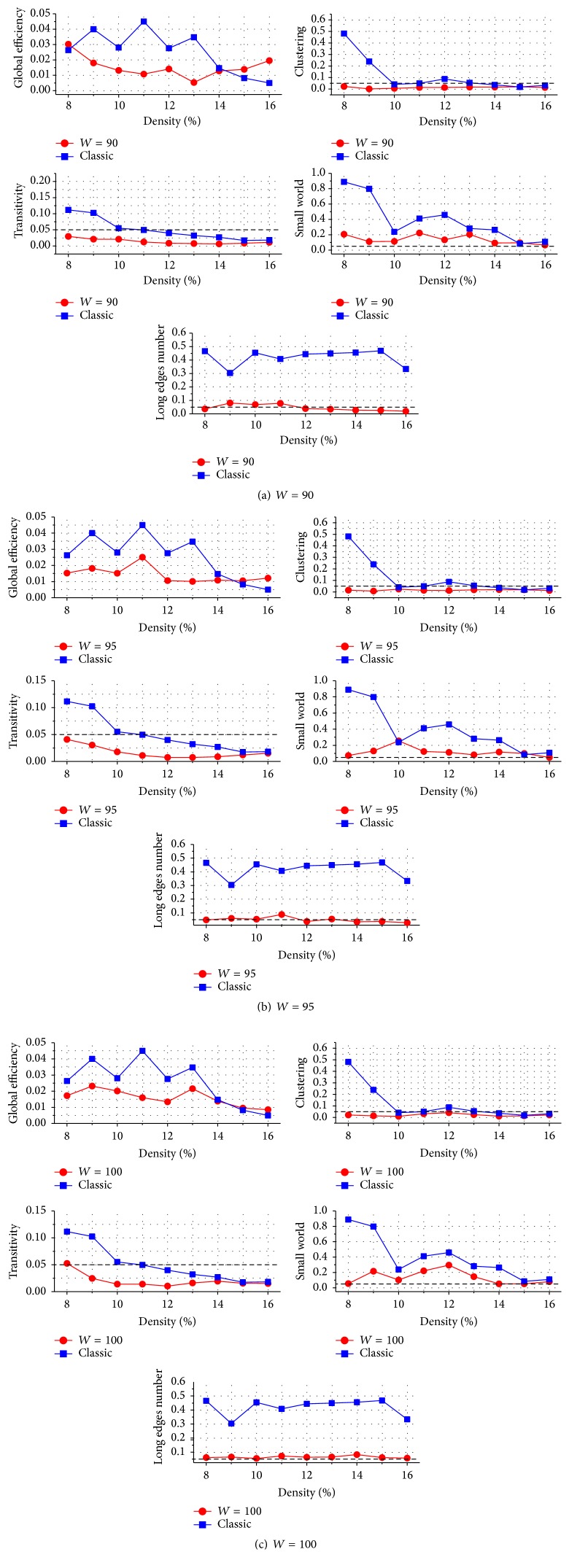
Statistical analysis of topological properties of the networks produced using the SNV (*W* = 90, 95, and 100) and classic methods. The blue and red lines represent *P* values obtained through the classic and SNV methods, respectively. Compared with that of the networks constructed by the classic method, *P* values of topological properties of the networks constructed by the SNV method significantly decreased (permutation test, *P* < 0.05 in efficiency, *P* < 0.01 in other properties).

We performed a *t*-test for the topological properties based on both network construction methods to compare the differences between the young and elderly groups.  Figure 4 shows the *P* values that were uncorrected for multiple comparison, which are convenient for straightforward comparison of magnitudes. Only 60 sets of network property comparisons in the classic method showed significant difference (*P* < 0.05). However, there were 92 sets showing significant difference in the SNV method, more than one and a half times that in classic method. Except for the small-world value and the number of long edges, 98.77% of the *P* values in SNV method showed significant difference (*P* < 0.05), indicating that the SNV method can identify much more statistically significant differences, which cannot be discovered when using the classic brain network construction method. Compared with the classic method, the *P* values of various topological properties by the SNV method significantly decreased in all of the three window widths (permutation test, *P* < 0.05 in efficiency, *P* < 0.01 in other properties). In our 135 sets of statistic comparisons, the *P* values of 124 sets (91.85%) decreased. In addition, 98.89% of the *P* values decreased at a network density range of 9–14%. When FDR correction was implemented for multiple comparisons at *q* value of 0.05, only 27 sets of comparisons showed significant difference in the classic method. However, there were 80 sets showing significant difference in the SNV method, nearly three times that in classic method.

### 3.3. Classification Results of the Young and Elderly Individuals Using SVM

We used the classic and SNV methods to build a functional brain network for each subject in the young and old groups, and then we attempted to use SVM as a classifier to classify the testing dataset into two groups. To analyze the impact of different window widths on classification, we performed classification experiment at *W* = 90, 95, and 100.

**Figure 5:**
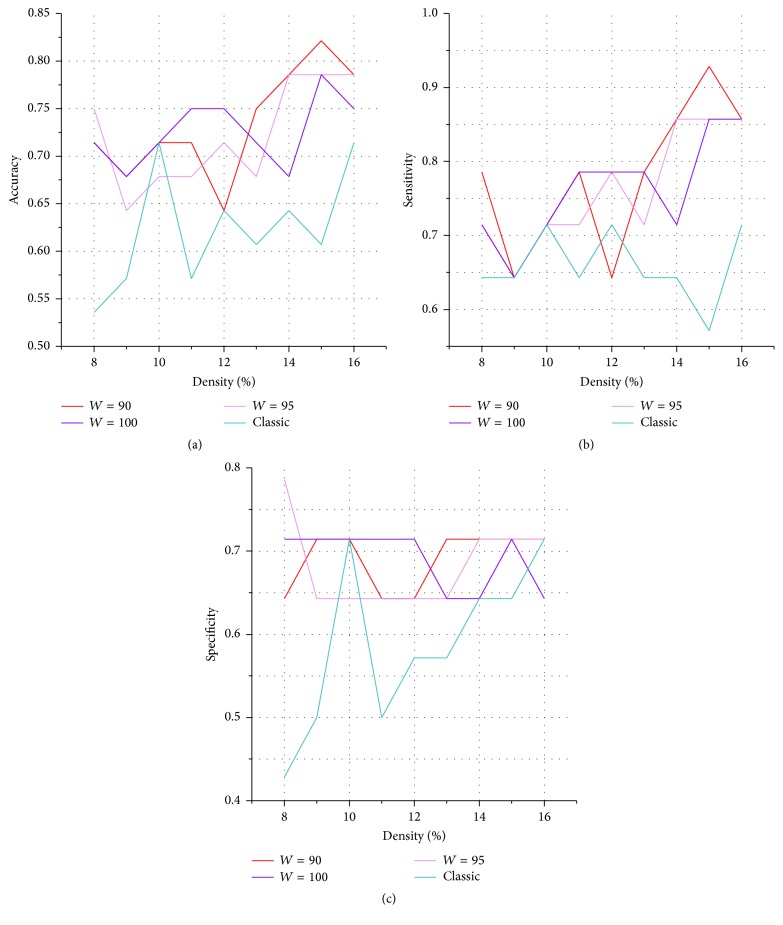
SVM classification results of young and elderly adults in the first feature set. Compared with classic network construction method, (a) accuracy, (b) sensitivity, and (c) specificity of classification based on the SNV method in the first feature set improved significantly.

Table 2 and Figure 5(a) show the accuracy rate of classification in the first set of classification features. The overall mean accuracy rate of the classic method was 62.3%. Compared with that classic method, the SNV method used in this study significantly improved the accuracy to 72.8% (permutation test, *P* < 0.01 in all of the three window widths). The highest accuracy was 82.1% (permutation test, *P* < 0.01; and the 95% confidence interval was 69.6–94.7%). Moreover, the sensitivity (Table 3 and Figure 5(b)) improved significantly from 65.9% to 76.7% (permutation test, *P* < 0.01 in all of the three window widths). The specificity (Table 4 and Figure 5(c)) also improved significantly from 58.7% to 68.8% (permutation test, *P* < 0.01 in all of the three window widths).

**Figure 6:**
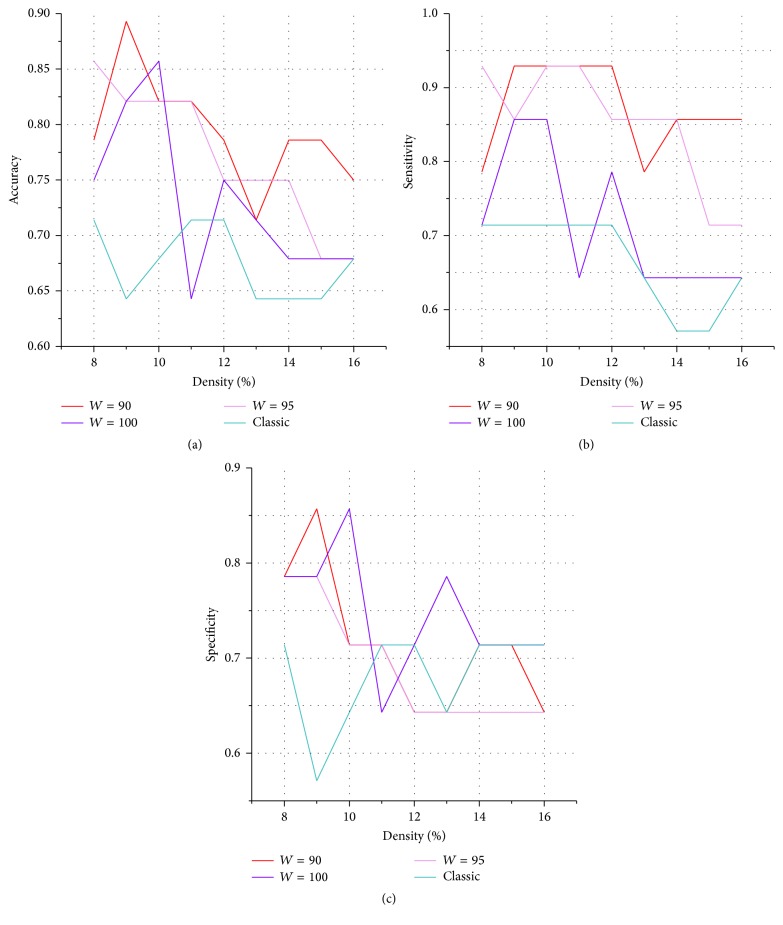
SVM classification results of young and elderly adults in the optimized feature set. Compared with classic network construction method, (a) accuracy, (b) sensitivity, and (c) specificity of classification based on the SNV method in the optimized feature set improved significantly.

The other set of classification features was optimized with nodal information, and the differences in node degree were prominent within parietal-(pre)motor, occipital, subcortical, and medial temporal systems (Table 1 and Figure 2) as revealed by the partition method based on a hierarchical clustering analysis as performed by Salvador et al. [37]. With the optimized feature set (Table 5 and Figure 6(a)), the overall mean accuracy rate of classification improved significantly from 67.5% in the classic method to 76.5% in the SNV method (permutation test, *P* < 0.001 at *W* = 90, 95; *P* < 0.05 at *W* = 100). In addition, the highest accuracy reached up to 89.3% (permutation test, *P* < 0.01; and the 95% confidence interval was 78.5–100%) when using the SNV method at *W* = 90 and density = 10%, which is consistent with or better than those obtained by the following similar studies: Vergun et al. [38] classified the young and elderly individuals based on the same dataset downloaded from 1000 Functional Connectomes Project with an accuracy of 84%. Supekar et al. [39] and Dosenbach et al. [40] reached an accuracy of 91% in classifying the children and adults by using the SVM method, respectively. Although these results were not that comparable because of the methodology and differences in raw data, they can still reflect to some extent the superiority of the SNV method. Furthermore, the sensitivity (Table 6 and Figure 6(b)) improved significantly from 66.7% to 81.2% (permutation test, *P* < 10*e* − 4 at *W* = 90, 95; no significant difference was observed at *W* = 100). The specificity (Table 7 and Figure 6(c)) improved significantly from 68.3% to 71.7% (permutation test, *P* < 0.01 at *W* = 100; no significant difference was observed at *W* = 90, 95).

## 4. Discussion

### 4.1. Differences in Topological Properties of Brain Network between the Young and Elderly Adults

The cognitive and memory functions of the brain generally decline with aging [41]. Different aspects of studies provide different explanations for this phenomenon. In terms of structural changes, previous studies have suggested that reduction in gray matter density and thinning of cortex may cause the decline in brain function during aging [5, 6]. With the recent development in graph theory and complex network, scientists have started to study aging and related diseases from the perspective of functional brain network, and they generally consider the decrease in cognitive and memory as a direct consequence of reduced efficiency and number of long edges in functional brain network during aging [3, 7–9, 15]. This present study observed the same results. From the perspective of physiology, this phenomenon is possibly caused by the changes and reconnection of the brain synapses.

Mean clustering coefficient is the average of clustering coefficient in all nodes in a network and indicates the extent of local cliquishness or local efficiency of information transfer [10, 42]. Numerous studies have demonstrated that brain networks evolved from an integrated system to a distributed one during aging [2, 14, 16], which is consistent with the observed increasing clustering coefficient in our study. Although both the old and young groups showed normal small-world architecture in the functional brain networks, increased clustering and decreased efficiency were found in elderly subjects, implying a degeneration process wherein the brain system shifted from a small-world network to a regular one along with normal aging [16, 26].

Transfer coefficient is the ratio of “triangles to triplets” in the network [43], from another aspect to measure the degree of tightness and easy exchange of information between network nodes. This study found that the transfer coefficient in the elderly group also increased to a certain degree. This finding is possibly caused by the increased linkages in the elderly group to maintain information transfer among different brain regions, which can be seen as an adaptive process of the brain during aging.

In this study, we found that the changes in the brain network properties during aging as revealed by the SNV method are consistent with those revealed by the classic method, indicating the validity of the SNV method. In other words, the SNV method did not result in false positive differences. By combining the previous results with ours, we consider that reorganizations occur in the brain network during aging, and differences do exist between the young and elderly groups on the level of functional brain network.

### 4.2. Analysis of *P* Values

This study calculated the topological properties and then performed statistical tests to compare the young and elderly groups. Similar experiments were performed in many previous studies. Sometimes studies capture statistically significant differences in some properties, whereas only nonsignificant differences were observed at times. For example, Wang et al. [7, 8] found reduction in global efficiency in the elderly group but did not observe significant differences in clustering coefficients. By contrast, significant differences in clustering coefficients were found in some other studies [2, 16]. One possible reason for this issue is that the approach employed in constructing brain networks cannot fully extract the “intrinsic edges” to avoid the useful signal being buried into noises. Our results showed that, under different network densities, the SNV method revealed much more significant differences, which cannot be discovered by the classic method. On this basis, the SNV method can better highlight the differences between the young and elderly groups according to various topological properties.

We also utilized another independent dataset consisting of nine AD patients and nine healthy controls to validate the performance of the SNV method (for more demographics and experiments, see Supplementary Materials available online at http://dx.doi.org/10.1155/2016/2429691). The brain networks were obtained in the same manner. The SNV method similarly revealed much more significant differences, and the *P* values of the network properties obtained using the SNV method significantly decreased compared with the classic method (Supplementary Figure 1).

These experimental results indicated that this novel method offers an apparent advantage in exploring significant alterations in aging or age-related diseases. This method reveals more differences, which the classic method cannot discover in brain network analysis, and thus this novel method is significant in exploring dynamic brain pathology.

### 4.3. SVM Classification

SVM is a machine learning method that can be trained and is widely used in nonlinear function classification. A good classification performance of the same kind of SVM reveals the consistency within group and the differences between groups. Our study used SVM to classify the samples into young group or elderly group. On the basis of both sets of features, the results showed that the accuracy, sensitivity, and specificity of the SNV method improved significantly in different sliding window widths compared with the classic method. That is, our SNV method can better highlight the differences between groups, making classification into two classes easier. Particularly for the optimized feature set, nodal information was combined with global network properties to improve classification accuracy. Given that the small-world value did not reveal significant difference at any density or any window width, which is consistent with the finding that a human brain exhibits small-world architecture in both the young and the elderly individuals [19], we removed this property from the feature set to avoid redundant characteristics. As we can see in Table 1, the nodes demonstrating significant difference were found in parietal-(pre)motor, occipital, subcortical, and medial temporal systems, which is consistent with a study by Chou et al. [44]. Moreover, St Jacques et al. [45] found an age-related difference in brain regions, such as the precentral area, for subsequent memory of negative stimuli, and Cao et al. [15] also found that brain regions showing significant age-related changes in weighted degree centrality were predominantly located in several default-mode regions. These regions exhibiting changes were also found in our results.

The highest accuracy in our study reached up to 89.3%. Compared with homologous researches, our method provided a preferable result based on a relatively small optimized feature set. In previous studies, Vergun et al. [38] reached 84% with 100 connection features, Supekar et al. [39] reached 91% with connectivity patterns between subcortical and primary sensory regions, and Dosenbach et al. [40] reached 91% with 200 functional connections.

This study obtained similar results from the independent dataset, indicating that our modeling method can fully extract effective characteristics from brain networks for classification. Furthermore, being a novel brain network construction method, the SNV method could help explore human normal aging or age-related diseases. Through the SNV method, the optimized mixed feature set can be possibly regarded as a remarkable biomarker, and an effective auxiliary diagnosis indicator based on SVM may be developed for future study or for clinical applications.

### 4.4. Impact of the Sliding Window Width on Brain Network Modeling

**Figure 7:**
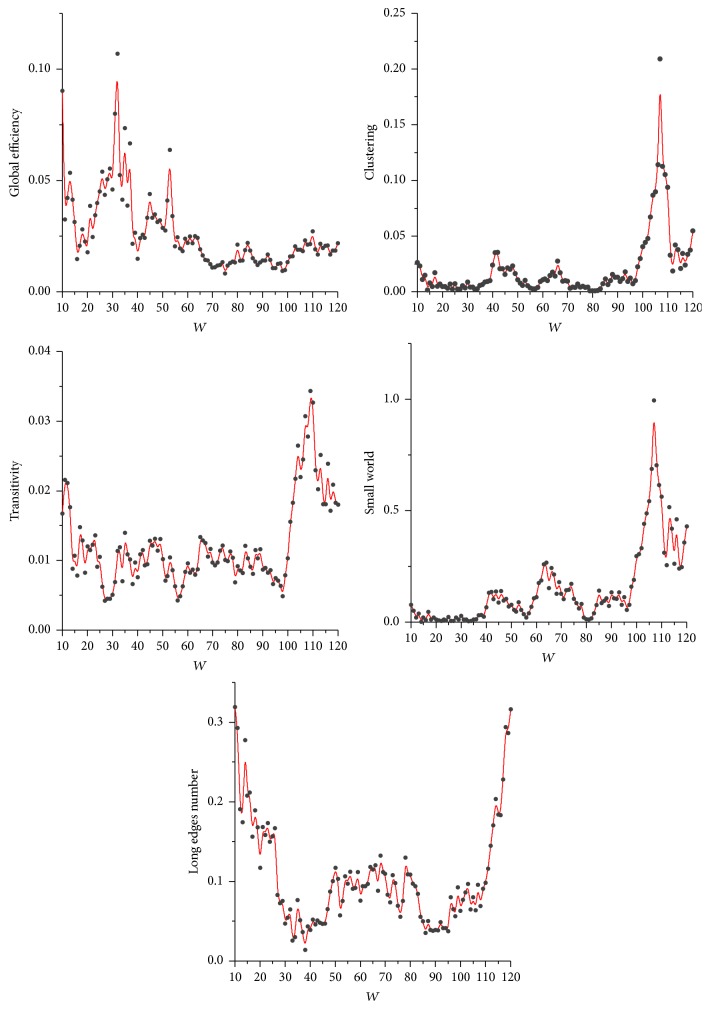
*P* values at window widths of 10–120. Dark gray dots represent *t*-test *P* values. Red solid lines represent B-spline-based nonparametric fits of *P* values. *P* values of all five network properties decreased to a relatively low level at a window width of 90–100.

This study used the sliding window widths of 90, 95, and 100 to establish the functional brain network of the young and the elderly groups. To analyze the impact of window width on our modeling approach, we constructed the brain networks by using the SNV method at window widths from 10 to 120 and calculated the *P* values of five network properties between groups. For easier visualization, we showed the results based on the middle density (12%) in Figure 7. At window widths below 90, the *P* values were generally increasing with decreasing window width. By contrast, at window width above 100, the overall performance rapidly deteriorated. Hence, the rational window width is 90–100.

Given that the TR of scans in our study was 2.0 s, window width of 90 equaled the scan duration of 3 min. In a review, Birn et al. [46] mentioned that many test-retest fMRI studies validated the reliability of functional connectivity using scan duration of 3–11 min and concluded that an increasing scan length can improve reproducibility. Van Dijk et al. [47] studied the intrinsic functional connectivity in human brain, and one of the most important conclusions was that correlation strengths can be stable at an acquisition time of as brief as 5 min. In addition, Gonzalez-Castillo et al. [48] suggested the use of longer scans above approximately 10 min. Although the context and ROIs of the above studies vary, they all suggested a relatively long but flexible scan duration of more than at least 3 min to achieve stable functional connectivity. Therefore, when the sliding window width is considerably narrow, the reliability of subnetworks constructed by Pearson correlation decreases, and thus the confidence of the SNV method declines. On this basis, subnetwork will be reliable when the scan duration of the subseries was longer than 3 min; that is, the window width should be above 90.

Moreover, although relatively stable brain connectivity can be acquired under a long scan duration, the performance of the brain network constructed by the classic method with full-length scans remained unsatisfactory because the effects of noise and individual difference are unavoidable during fMRI acquisition. For example, two uncorrelated time courses can be quite correlated when noise is added to time courses, resulting in a spurious edge in the network. We therefore introduced “voting” when constructing brain network to promote the discriminability of identifying a spurious edge. That is to say, “voting,” to a large extent, reduces the false positive rate of identifying an edge. In this way, we can obtain the most intrinsic edges among those “stable edges.” The brain network composed of these intrinsic edges can improve the consistency within group and highlight the differences between groups. Given this, when the sliding window width is considerably wide, the number of subnetworks will be considerably small in our modeling, and the active effect of the final voting process will be reduced. The experimental results demonstrated this inference. Figure 7 suggests that better modeling results will be obtained when the window width is lower than 100, which provides sufficient number of subnetworks for voting. In this case, the number of the subnetworks was maintained above 25. The conclusion was also supported by the supplementary dataset.

### 4.5. Limitations

Some limitations in our SNV method must be clarified. First, our method can only be applied to dataset that contains a sequence of elements, such as BOLD time course from fMRI, and is not appropriate when applied to single-variable dataset, such as cortex thickness from structural MRI, in which case an individual network cannot be constructed by correlation method. We therefore cannot split a raw data sequence into windowed subsequences. Second, our classification feature lacks mechanistic interpretability. Although a simple or low-dimensional feature in our study can greatly save calculating resources and avoid overfitting, it in turn cannot comprehensively represent the whole brain network, indicating that we cannot figure out the connectivity by nodal information in feature vector. Third, the selection of window width in window-sliding method is an open issue in recent years [49, 50]. We usually choose a window width through the rule of thumb to avoid spurious fluctuation in network state [50]. Although our window widths were verified by experimental results, a data-driven method is urgently needed to address this issue, and that will be one of our studies in the future.

## Supplementary Material

The supplementary Material includes the definitions of the network properties and the experimental results of the independent cohort. These results are consistent with the results of the primary cohort.

## Figures and Tables

**Table 1:** Regions showing significant difference in node degree.

Region	Hemisphere	AAL ID	Cluster
Precentral gyrus	Left	1	Parietal-(pre)motor
Superior occipital gyrus	Left	49	Occipital
Thalamus	Left	77	Subcortical
	Right	78	Subcortical
Temporal pole, superior temporal gyrus	Right	84	Medial temporal

The last column exhibits the clusters which the region belonged to according to the hierarchical clustering analysis in a previous study [37].

**Table 2:** SVM accuracy in the first feature set.

Density	8%	9%	10%	11%	12%	13%	14%	15%	16%
*W*									
90	0.714	0.679	0.714	0.714	0.643	0.750	0.786	0.821	0.786
95	0.750	0.643	0.679	0.679	0.714	0.679	0.786	0.786	0.786
100	0.714	0.679	0.714	0.750	0.750	0.714	0.679	0.786	0.750
Classic	0.536	0.571	0.714	0.571	0.643	0.607	0.643	0.607	0.714

Accuracy was significantly higher in the SNV method than in the classic method (permutation test, *P* < 0.01 in all of the three window widths).

**Table 3:** SVM sensitivity in the first feature set.

Density	8%	9%	10%	11%	12%	13%	14%	15%	16%
*W*									
90	0.786	0.643	0.714	0.786	0.643	0.786	0.857	0.929	0.857
95	0.714	0.643	0.714	0.714	0.786	0.714	0.857	0.857	0.857
100	0.714	0.643	0.714	0.786	0.786	0.786	0.714	0.857	0.857
Classic	0.643	0.643	0.714	0.643	0.714	0.643	0.643	0.571	0.714

Sensitivity was significantly higher in the SNV method than in the classic method (permutation test, *P* < 0.01 in all of the three window widths).

**Table 4:** SVM specificity in the first feature set.

Density	8%	9%	10%	11%	12%	13%	14%	15%	16%
*W*									
90	0.643	0.714	0.714	0.643	0.643	0.714	0.714	0.714	0.714
95	0.786	0.643	0.643	0.643	0.643	0.643	0.714	0.714	0.714
100	0.714	0.714	0.714	0.714	0.714	0.643	0.643	0.714	0.643
Classic	0.429	0.500	0.714	0.500	0.571	0.571	0.643	0.643	0.714

Specificity was significantly higher in the SNV method than in the classic method (permutation test, *P* < 0.01 in all of the three window widths).

**Table 5:** SVM accuracy in the optimized feature set.

Density	8%	9%	10%	11%	12%	13%	14%	15%	16%
*W*									
90	0.786	0.893	0.821	0.821	0.786	0.714	0.786	0.786	0.750
95	0.857	0.821	0.821	0.821	0.750	0.750	0.750	0.679	0.679
100	0.750	0.821	0.857	0.643	0.750	0.714	0.679	0.679	0.679
Classic	0.714	0.643	0.679	0.714	0.714	0.643	0.643	0.643	0.679

Accuracy was significantly higher in the SNV method than in the classic method (permutation test, *P* < 0.001 at *W* = 90,95; *P* < 0.05 at *W* = 100).

**Table 6:** SVM sensitivity in the optimized feature set.

Density	8%	9%	10%	11%	12%	13%	14%	15%	16%
*W*									
90	0.786	0.929	0.929	0.929	0.929	0.786	0.857	0.857	0.857
95	0.929	0.857	0.929	0.929	0.857	0.857	0.857	0.714	0.714
100	0.714	0.857	0.857	0.643	0.786	0.643	0.643	0.643	0.643
Classic	0.714	0.714	0.714	0.714	0.714	0.643	0.571	0.571	0.643

Sensitivity was significantly higher in the SNV method than in the classic method (permutation test, *P* < 10*e* − 4 at *W* = 90,95; no significant difference was observed at *W* = 100).

**Table 7:** SVM specificity in the optimized feature set.

Density	8%	9%	10%	11%	12%	13%	14%	15%	16%
*W*									
90	0.786	0.857	0.714	0.714	0.643	0.643	0.714	0.714	0.643
95	0.786	0.786	0.714	0.714	0.643	0.643	0.643	0.643	0.643
100	0.786	0.786	0.857	0.643	0.714	0.786	0.714	0.714	0.714
Classic	0.714	0.571	0.643	0.714	0.714	0.643	0.714	0.714	0.714

Specificity was significantly higher in the SNV method than in the classic method at one window width (permutation test, *P* < 0.01 at *W* = 100; no significant difference was observed at *W* = 90,95).
