# Supplementary material for: A Novel Brain Network Construction Method for Exploring Age-Related Functional Reorganization
Source: Comput Intell Neurosci. 2016 Feb 29;2016:2429691. doi: 10.1155/2016/2429691 (PMC4789383; doi:10.1155/2016/2429691)
Supplement: Supplementary file 1 — The supplementary Material includes the definitions of the network properties and the experimental results of the independent cohort. These results are consistent with the results of the primary cohort. [file 2429691.f1.pdf]

Supplementary materials

S1. Network properties

The global efficiency is defined as:

$$\mathrm{Eg} = \frac{1}{N(N-1)}\sum_{i\neq j}\frac{1}{dis_{i,j}}$$

where N is the total number of nodes in a network, and $dis_{i,j}$ is the shortest path length between nodes i and j.

The nodal clustering coefficient of node i is defined as:

$$C_i = \frac{t_i}{k_i(k_i-1)/2}$$

where k_i is the number of immediate neighbors of node i, or degree of node i, and t_i is the number of edges among the immediate neighbors. The mean clustering coefficient, or clustering coefficient, is the average value of nodal clustering coefficient of all nodes:

$$\mathrm{C} = \frac{1}{N}\sum C_i$$

Transitivity is another form of clustering coefficient, which is defined as:

$$\mathrm{I} = \frac{\sum_i 2t_i}{\sum_i k_i(k_i-1)}$$

The small-world value is defined as the ratio of mean clustering coefficient and character path length:

$$\mathrm{S} = \mathrm{C}/\mathrm{L}$$

Long edge number is defined as the total number of long edges in a network. A long edge is defined as an edge whose anatomic distance of its two nodes is greater than 75 mm (He et al., 2007).

S2. Experiments on the independent cohort

A total of 9 right-handed AD patients (4 males, 5 females; mean age: 65.78 years old;

range: 55-79 years old) and 9 right-handed healthy controls (4 males, 5 females; mean age: 64.88 years old; range: 55-81 years old) were recruited to participate as an independent dataset for model evaluation in this research, which was approved by the internal Institutional Review Board of Tongji Hospital. Informed consents were obtained from all participants.

The raw time courses include 230 time points, TR=2.0 s. According to the criterion presented in Section 4.4, the selected window widths were 180, 190, and 200. The P values of the properties of the network constructed using the SNV method apparently decreased compared with the classic method among most density ranges (Supplementary Figure 1).

With the first feature set described in section 2.5, the accuracy, sensitivity, and specificity of classification obviously improved compared to the classic method (Supplementary Figure 2). The highest accuracy was 88.9% (permutation test, $P < 0.05$; and the 95% confidence interval was [75.3-100%]).

With the optimized feature set, Supramarginal gyrus (AAL ID: 63) and Inferior temporal gyrus (AAL ID: 90) were found to be significantly different in nodal degree. The accuracy, sensitivity, and specificity of classification were shown in Supplementary Figure 3, and the highest accuracy reached up to 94.4% (permutation test, $P < 0.05$; and the 95% confidence interval was [84.2-100%]).

Reference

He Y, Chen ZJ, Evans AC. Small-World Anatomical Networks in the Human Brain Revealed by Cortical Thickness from MRI. Cereb Cortex. 2007 2007-10-01;17(10):2407-19.

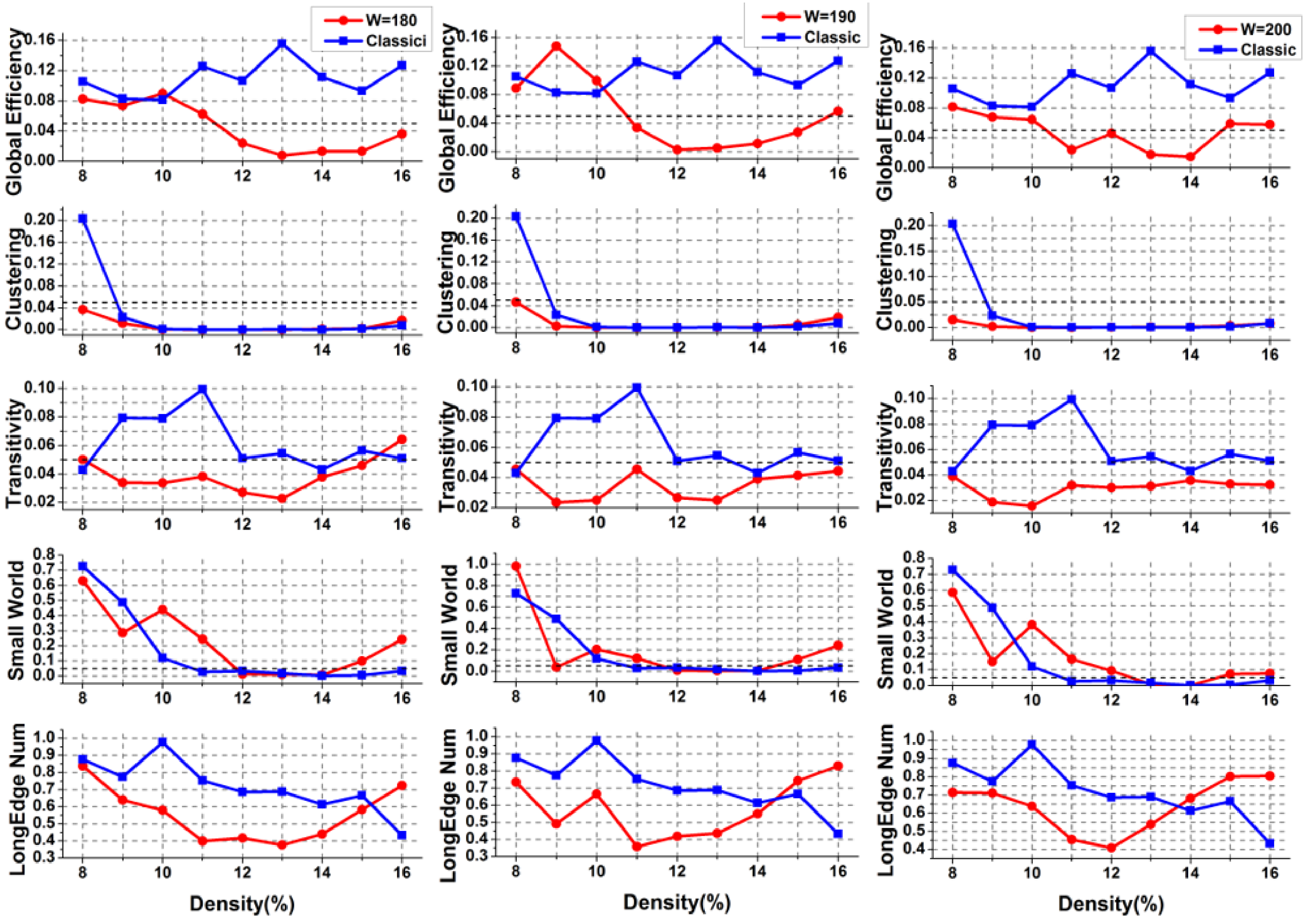

(A) W=180

(B) W=190

(C) W=200

Supplementary Figure 1

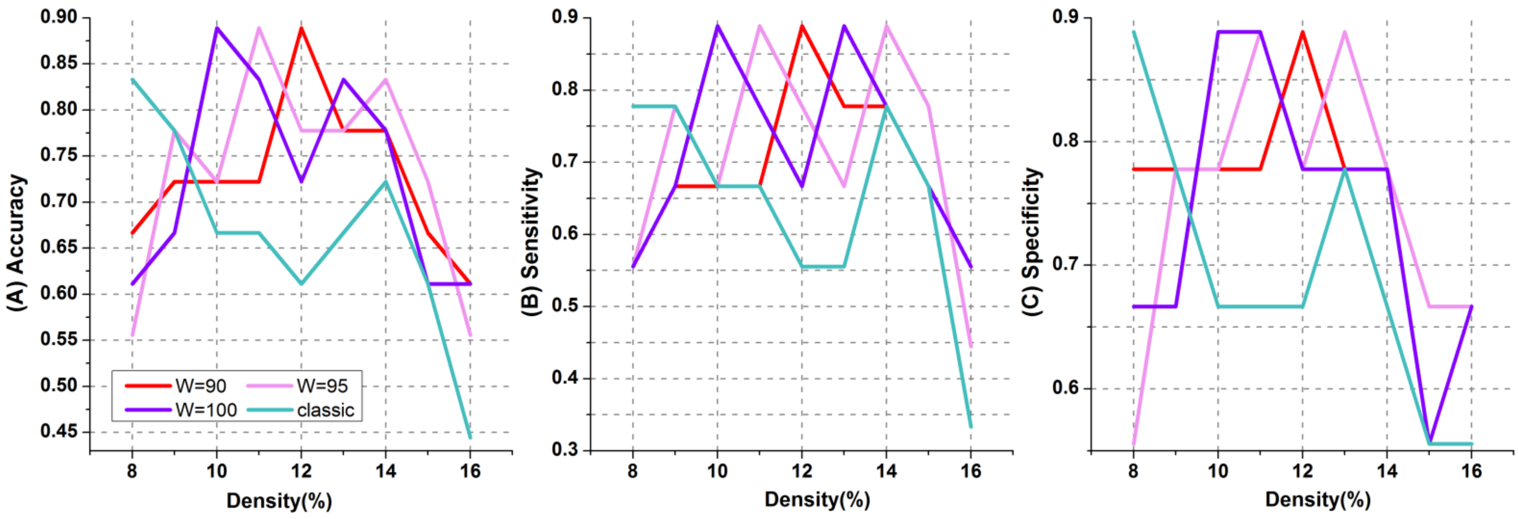

Supplementary Figure 2. (A)accuracy, (B)sensitivity, and (C) specificity in the first feature set

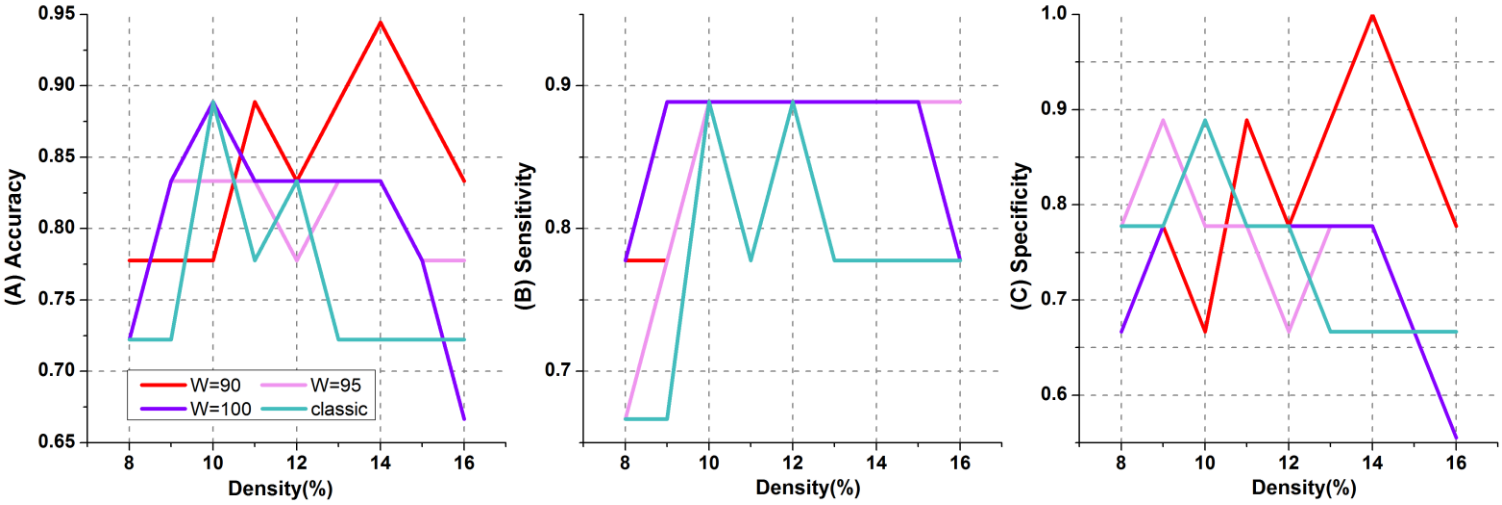

Supplementary Figure 3. (A)accuracy, (B)sensitivity, and (C) specificity in the optimized feature set
